# Supplementary material for: Effect of hysterectomy on the risk of ovarian cancer: A South Korean national cohort study
Source: PLoS One. 2026 May 6;21(5):e0348127. doi: 10.1371/journal.pone.0348127 (PMC13148655; doi:10.1371/journal.pone.0348127)
Supplement: S1 Table — (DOCX) [file pone.0348127.s001.docx]

| Supplementary table 1.Characteristics of the hysterectomy and non-hysterectomy groups among the subjects in this study (before propensity matching) | | | | | | |
| --- | --- | --- | --- | --- | --- | --- |
|  |  |  |  |  |  |  |
|  | Non-Hysterectomy | Hysterectomy | Total | P-value | Standardized mean difference | Missing, % |
| Nunber of women | 885,544 | 30,786 | 916,330 |  |  |  |
| Follow-up period (years) | 12.6 [10.8-14.6] | 13.4 [11.1-15.8] | 12.6 [10.8-14.6] | <0.001 | 0.265 | 0 |
| Median age (years) | 48 [42-52] | 47 [44-49] | 48 [42-52] | <0.001 | 0.198 | 0 |
| Age at inclusion (years) |  |  |  | <0.001 | 0.617 | 0 |
| 40~44 | 328,942 (37.1) | 9,532 (31) | 338,474 (36.9) |  |  |  |
| 45~49 | 196,358 (22.2) | 14,162 (46) | 210,520 (23) |  |  |  |
| 50~55 | 225,241 (25.4) | 6,094 (19.8) | 231,335 (25.2) |  |  |  |
| 55~60 | 135,003 (15.2) | 998 (3.2) | 136,001 (14.8) |  |  |  |
| Year at inclusion |  |  |  | <0.001 | 0.255 | 0 |
| 2003~2005 | 211,452 (23.9) | 10,856 (35.3) | 222,308 (24.3) |  |  |  |
| 2006~2008 | 356,775 (40.3) | 10,073 (32.7) | 366,848 (40) |  |  |  |
| 2009~2011 | 317,317 (35.8) | 9,857 (32) | 327,174 (35.7) |  |  |  |
| Median BMI (kg/m^2^) | 23.2 [21.4-25.3] | 23.7 [21.9-25.9] | 23.2 [21.4-25.4] | <0.001 | 0.112 | 0 |
| BMI (kg/m2) |  |  |  | <0.001 | 0.157 | 0 |
| <18.5 | 21,944 (2.5) | 473 (1.5) | 22,417 (2.4) |  |  |  |
| 18.5-22.9 | 389,677 (44) | 11,658 (37.9) | 401,335 (43.8) |  |  |  |
| 23-24.9 | 217,101 (24.5) | 7,958 (25.8) | 225,059 (24.6) |  |  |  |
| 25-29.9 | 225,051 (25.4) | 9,228 (30) | 234,279 (25.6) |  |  |  |
| ≥30 | 31,771 (3.6) | 1,469 (4.8) | 33,240 (3.6) |  |  |  |
| SES |  |  |  | <0.001 | 0.058 | 0 |
| Mid~high SES | 882,286 (99.6) | 30,536 (99.2) | 912,822 (99.6) |  |  |  |
| Low SES | 3,258 (0.4) | 250 (0.8) | 3,508 (0.4) |  |  |  |
| Region |  |  |  | 0.74 | 0.003 | 0 |
| Urban area | 259,843 (29.3) | 9,061 (29.4) | 268,904 (29.3) |  |  |  |
| Rural area | 625,701 (70.7) | 21,725 (70.6) | 647,426 (70.7) |  |  |  |
| CCI |  |  |  | <0.001 | 0.071 | 0 |
| 0 | 736,748 (83.2) | 25,087 (81.5) | 761,835 (83.1) |  |  |  |
| 1 | 123,675 (14) | 4,453 (14.5) | 128,128 (14) |  |  |  |
| ≥2 | 25,121 (2.8) | 1,246 (4) | 26,367 (2.9) |  |  |  |
| Parity |  |  |  | <0.001 | 0.255 | 0 |
| 0 or not respond | 199,494 (22.5) | 4,463 (14.5) | 203,957 (22.3) |  |  |  |
| 1 | 87,910 (9.9) | 3,793 (12.3) | 91,703 (10) |  |  |  |
| 2 | 525,612 (59.4) | 20,925 (68) | 546,537 (59.6) |  |  |  |
| ≥3 | 72,528 (8.2) | 1,605 (5.2) | 74,133 (8.1) |  |  |  |
| Age at menarche (years) |  |  |  | <0.001 | 0.095 | 2 |
| <13 | 217,190 (25.1) | 6,345 (20.9) | 223,535 (24.9) |  |  |  |
| ≥13 | 649,312 (74.9) | 24,080 (79.1) | 673,392 (75.1) |  |  |  |
| Menopause before inclusion |  |  |  | <0.001 | 0.171 | 0 |
| Absent | 616,327 (69.6) | 23,780 (77.2) | 640,107 (69.9) |  |  |  |
| Present | 269,217 (30.4) | 7,006 (22.8) | 276,223 (30.1) |  |  |  |
| Smoking |  |  |  | 0.873 | 0.002 | 8 |
| Never | 779,018 (94.4) | 12,618 (94.3) | 791,636 (94.4) |  |  |  |
| Past | 12,876 (1.6) | 212 (1.6) | 13,088 (1.6) |  |  |  |
| Current | 33,566 (4.1) | 555 (4.1) | 34,121 (4.1) |  |  |  |
| Alcohol (per week) |  |  |  | <0.001 | 0.099 | 8 |
| None | 619,657 (74.6) | 9,495 (70.3) | 629,152 (74.5) |  |  |  |
| ~2/week | 198,067 (23.8) | 3,686 (27.3) | 201,753 (23.9) |  |  |  |
| 3~6/week | 8,994 (1.1) | 240 (1.8) | 9,234 (1.1) |  |  |  |
| Daily | 4,209 (0.5) | 85 (0.6) | 4,294 (0.5) |  |  |  |
| Physical exercise (per week) |  |  |  | 0.009 | 0.039 | 8 |
| None | 534,651 (64.5) | 8,672 (64.3) | 543,323 (64.5) |  |  |  |
| 1~2 | 155,705 (18.8) | 2,602 (19.3) | 158,307 (18.8) |  |  |  |
| 3~4 | 79,712 (9.6) | 1,331 (9.9) | 81,043 (9.6) |  |  |  |
| 5~6 | 24,758 (3) | 408 (3) | 25,166 (3) |  |  |  |
| Daily | 34,260 (4.1) | 480 (3.6) | 34,740 (4.1) |  |  |  |
| DM before inclusion |  |  |  | 0.025 | 0.006 | 0 |
| Absent | 807,813 (91.2) | 28,197 (91.6) | 836,010 (91.2) |  |  |  |
| Present | 77,731 (8.8) | 2,589 (8.4) | 80,320 (8.8) |  |  |  |
| Hypertension before inclusion |  |  |  | 0.038 | 0.025 | 0 |
| Absent | 740,330 (83.6) | 25,600 (83.2) | 765,930 (83.6) |  |  |  |
| Present | 145,214 (16.4) | 5,186 (16.8) | 150,400 (16.4) |  |  |  |
| Dyslipidemia before inclusion |  |  |  | <0.001 | 0.015 | 0 |
| Absent | 775,489 (87.6) | 27,166 (88.2) | 802,655 (87.6) |  |  |  |
| Present | 110,055 (12.4) | 3,620 (11.8) | 113,675 (12.4) |  |  |  |
| MHT before inclusion |  |  |  | <0.001 | 0.172 | 0 |
| Absent | 851,913 (96.2) | 30,451 (98.9) | 882,364 (96.3) |  |  |  |
| Present | 33,631 (3.8) | 335 (1.1) | 33,966 (3.7) |  |  |  |
| Adnexal surgery before inclusion |  |  |  | 0.815 | 0.002 | 0 |
| Absent | 884,957 (99.9) | 30,764 (99.9) | 915,721 (99.9) |  |  |  |
| Present | 587 (0.1) | 22 (0.1) | 609 (0.1) |  |  |  |
| Uterine fibroids before inclusion |  |  |  | <0.001 | 1.788 | 0 |
| Absent | 839,137 (94.8) | 9,125 (29.6) | 848,262 (92.6) |  |  |  |
| Present | 46,407 (5.2) | 21,661 (70.4) | 68,068 (7.4) |  |  |  |
| Endometriosis before inclusion |  |  |  | <0.001 | 0.543 | 0 |
| Absent | 875,958 (98.9) | 26,159 (85) | 902,117 (98.4) |  |  |  |
| Present | 9,586 (1.1) | 4,627 (15) | 14,213 (1.6) |  |  |  |
|  |  |  |  |  |  |  |
| DM, diabetes mellitus; CCI, Charlson comorbidity index; MHT, menopausal hormone therapy; SES, socioeconomic status | | | | | | |
| The data is presented as either a number and percentage, or as a median with the interquartile range. | | | | | | |
