# Supplementary material for: Effect of hysterectomy on the risk of ovarian cancer: A South Korean national cohort study
Source: PLoS One. 2026 May 6;21(5):e0348127. doi: 10.1371/journal.pone.0348127 (PMC13148655; doi:10.1371/journal.pone.0348127)
Supplement: S2 Table — (DOCX) [file pone.0348127.s002.docx]

| Supplementary Table 2. Exploratory risk of ovarian cancer by age in women who had a hysterectomy using Cox proportional hazards analysis. | | | | |
| --- | --- | --- | --- | --- |
|  |  |  |  |  |
|  | 40~49 years | | 50~59 years | |
|  | HR (95% CI) ^a^ | P-value | HR (95% CI) ^a^ | P-value |
| Ovarian cancer |  |  |  |  |
| Hysterectomy (with/without concomitant adnexal surgery) | 1.167 (0.54-2.522) | 0.695 | 1.143 (0.414-3.152) | 0.796 |
| Hysterectomy without concomitant adnexal surgery | 1.182 (0.529-2.638) | 0.683 | 1 (0.323-3.101) | 1 |
| Hysterectomy with concomitant adnexal surgery | 1 (0.063-15.988) | 1 | 2 (0.181-22.056) | 0.571 |
|  |  |  |  |  |
| CI, confidence interval; HR, hazard ratio | | | | |
| ^a^ Because propensity score matching was performed, hazard ratios were not adjusted for additional confounders. | | | | |
