# Supplementary material for: Effect of hysterectomy on the risk of ovarian cancer: A South Korean national cohort study
Source: PLoS One. 2026 May 6;21(5):e0348127. doi: 10.1371/journal.pone.0348127 (PMC13148655; doi:10.1371/journal.pone.0348127)
Supplement: S3 Table — (DOCX) [file pone.0348127.s003.docx]

| **Supplementary Table 2. Analysis of ovarian cancer incidence per 100,000 person-years in women with and without hysterectomy using Korean National Health Insurance data from 2002-2020.** | | |
| --- | --- | --- |
|  |  |  |
|  | Non-Hysterectomy | Hysterectomy |
| Total | 20/154,403 (13) | 28/154,109 (18) |
| Age at inclusion (years) |  |  |
| 40~44 | 2/39,279 (5) | 8/36,745 (22) |
| 45~49 | 10/62,695 (16) | 10/74,809 (13) |
| 50~54 | 8/41,805 (19) | 9/35,484 (25) |
| 55~59 | 0/10,624 (0) | 1/7,071 (14) |
| Year at inclusion |  |  |
| 2003~2005 | 1/22,224 (4) | 6/23,881 (25) |
| 2006~2008 | 9/59,603 (15) | 8/56,314 (14) |
| 2009~2011 | 10/72,577 (14) | 14/73,915 (19) |
| BMI (kg/m2) |  |  |
| <18.5 | 0/2,756 (0) | 1/2,244 (45) |
| 18.5-22.9 | 7/60,142 (12) | 7/61,501 (11) |
| 23-24.9 | 4/38,962 (10) | 7/39,792 (18) |
| 25-29.9 | 7/45,865 (15) | 12/44,369 (27) |
| ≥30 | 2/6,679 (30) | 1/6,203 (16) |
| SES |  |  |
| Mid~high SES | 20/153,922 (13) | 28/153,385 (18) |
| Low SES | 0/481 (0) | 0/724 (0) |
| Region |  |  |
| Urban area | 4/44,472 (9) | 8/43,820 (18) |
| Rural area | 16/109,932 (15) | 20/110,290 (18) |
| CCI |  |  |
| 0 | 16/125,206 (13) | 25/125,589 (20) |
| 1 | 3/23,926 (13) | 2/22,901 (9) |
| ≥2 | 1/5,271 (19) | 1/5,619 (18) |
| Parity |  |  |
| 0 or not respond | 7/26,906 (26) | 5/26,570 (19) |
| 1 | 2/18,893 (11) | 2/17,821 (11) |
| 2 | 9/99,360 (9) | 18/100,683 (18) |
| ≥3 | 2/9,246 (22) | 3/9,036 (33) |
| Age at menarche (years) |  |  |
| <13 | 6/33,674 (18) | 6/34,170 (18) |
| ≥13 | 14/120,730 (12) | 22/119,939 (18) |
| Menopause before inclusion |  |  |
| Absent | 19/129,408 (15) | 25/130,301 (19) |
| Present | 1/24,995 (4) | 3/23,809 (13) |
| Smoking |  |  |
| Never | 20/144,757 (14) | 27/145,488 (19) |
| Past | 0/2,764 (0) | 1/2,295 (44) |
| Current | 0/6,883 (0) | 0/6,326 (0) |
| Alcohol (per week) |  |  |
| None | 18/106,401 (17) | 22/108,987 (20) |
| ~2/week | 2/44,702 (4) | 6/41,353 (15) |
| 3~6/week | 0/2,127 (0) | 0/2,826 (0) |
| Daily | 0/1,174 (0) | 0/945 (0) |
| Physical exercise (per week) |  |  |
| None | 18/96,770 (19) | 18/98,049 (18) |
| 1~2 | 1/31,171 (3) | 6/29,896 (20) |
| 3~4 | 0/15,722 (0) | 3/15,688 (19) |
| 5~6 | 1/4,983 (20) | 0/4,684 (0) |
| Daily | 0/5,758 (0) | 1/5,793 (17) |
| DM before inclusion |  |  |
| Absent | 15/138,745 (11) | 24/139,152 (17) |
| Present | 5/15,659 (32) | 4/14,957 (27) |
| Hypertension before inclusion |  |  |
| Absent | 15/125,057 (12) | 21/125,168 (17) |
| Present | 5/29,347 (17) | 7/28,941 (24) |
| Dyslipidemia before inclusion |  |  |
| Absent | 15/130,598 (11) | 27/131,151 (21) |
| Present | 5/23,805 (21) | 1/22,959 (4) |
| MHT before inclusion |  |  |
| Absent | 19/150,778 (13) | 28/152,192 (18) |
| Present | 1/3,626 (28) | 0/1,918 (0) |
| Adnexal surgery before inclusion |  |  |
| Absent | 20/153,974 (13) | 28/153,989 (18) |
| Present | 0/430 (0) | 0/120 (0) |
| Uterine fibroids before inclusion |  |  |
| Absent | 5/39,789 (13) | 9/40,406 (22) |
| Present | 15/114,614 (13) | 19/113,703 (17) |
| Endometriosis before inclusion |  |  |
| Absent | 18/130,059 (14) | 25/129,124 (19) |
| Present | 2/24,344 (8) | 3/24,985 (12) |
|  |  |  |
| DM, diabetes mellitus; CCI, Charlson comorbidity index; MHT, menopausal hormone therapy; SES, socioeconomic status | | |
| The incidence rates are expressed as ovarian cancer events per 100,000 person-years of observation. | | |
