# Supplementary material for: Effect of hysterectomy on the risk of ovarian cancer: A South Korean national cohort study
Source: PLoS One. 2026 May 6;21(5):e0348127. doi: 10.1371/journal.pone.0348127 (PMC13148655; doi:10.1371/journal.pone.0348127)
Supplement: Attachment — Submitted filename: New Microsoft Word Document.docx [file pone.0348127.s004.docx]

**Critical Appraisal of Manuscript PONE-D-25-36133: Effect of Hysterectomy on the Risk of Ovarian Cancer**

This manuscript addresses a clinically significant and relevant question: the association between hysterectomy and the subsequent risk of ovarian cancer, focusing on a South Korean national cohort. The study's objective to investigate this relationship within an understudied Asian population, utilizing a large, population-based database, is commendable and represents a valuable contribution to the field. The use of the Korean National Health Insurance Service (NHIS) database provides a powerful foundation for longitudinal research.

However, despite the important research question and the potential of the dataset, the study is undermined by two fundamental and critical flaws in its methodology and interpretation. These flaws are of sufficient magnitude to invalidate the study's primary conclusions.

First, there is a **critical methodological failure in the application of propensity score matching (PSM)**. The central analytical strategy for controlling confounding—the very basis for ensuring the comparability of the hysterectomy and non-hysterectomy groups—demonstrably failed to achieve its objective. As presented in the manuscript's own results, numerous key covariates, including age, BMI, and several pre-existing medical conditions, remain severely imbalanced between the two groups after matching. This failure compromises the study's internal validity and means that any observed association, or lack thereof, cannot be reliably attributed to hysterectomy, as it is likely biased by residual confounding.

Second, the study suffers from **insufficient statistical power**, a consequence of a low number of outcome events (48 incident ovarian cancer cases). This low event rate has resulted in a highly imprecise effect estimate for the primary outcome (Hazard Ratio 1.42, 95% Confidence Interval [CI] 0.79-2.56). The extremely wide confidence interval, which encompasses both a potential 21% risk reduction and a 156% risk increase, renders the result inconclusive. The authors' firm conclusion of "no significant association" is an overstatement that misinterprets statistical non-significance in an underpowered study as definitive evidence of no effect.

In its current form, the manuscript does not provide a reliable answer to its research question. The conclusions are not supported by the presented analysis due to uncontrolled confounding and profound statistical uncertainty. Therefore, the manuscript is not recommended for publication without a substantial and fundamental re-analysis of the data and a complete revision of its interpretation and conclusions.

**Major Methodological and Statistical Concerns**

**Compromised Internal Validity due to Failed Covariate Balancing**

The analytical framework of this retrospective cohort study is entirely dependent on the successful implementation of propensity score matching (PSM). In observational research where treatment is not randomized, PSM is employed to create treatment and control groups that are comparable on a wide range of observed baseline characteristics, thereby reducing bias from confounding variables and mimicking the conditions of a randomized controlled trial. The authors correctly identified this as the necessary approach for their study design. However, the data presented in the manuscript unequivocally demonstrate that this crucial methodological step was unsuccessful.

The evidence of this failure is starkly presented in Table 1 of the manuscript, which details the characteristics of the study groups *after* the 1:1 propensity score matching was performed. A successful matching procedure should result in groups that are well-balanced, with no statistically significant differences in the distribution of the covariates used for matching. Contrary to this expectation, Table 1 reveals persistent, statistically significant imbalances across numerous key prognostic and demographic factors.

The following table summarizes the most concerning imbalances reported by the authors, which directly contradict the purpose of the matching procedure.

**Table 1: Summary of Significantly Imbalanced Covariates After Propensity Score Matching (Data from Manuscript Table 1)**

| Covariate | Non-Hysterectomy Group (N=13,059) | Hysterectomy Group (N=13,059) | P-value | Standardized Mean Difference (SMD) |
| --- | --- | --- | --- | --- |
| **Age at inclusion (years)** | Distribution differs significantly | Distribution differs significantly | <0.001 | 0.174 |
| **Year at inclusion** | Distribution differs significantly | Distribution differs significantly | <0.001 | 0.041 |
| **BMI (kg/m2)** | Distribution differs significantly | Distribution differs significantly | <0.001 | 0.041 |
| **Menopause before inclusion** | 1,992 (15.3%) | 1,869 (14.3%) | <0.001 | 0.027 |
| **MHT before inclusion** | 300 (2.3%) | 168 (1.3%) | <0.001 | 0.076 |
| **Adnexal surgery before inclusion** | 39 (0.3%) | 11 (0.1%) | <0.001 | 0.049 |
| **Uterine fibroids before inclusion** | 9,776 (74.9%) | 9,705 (74.3%) | <0.001 | 0.012 |
| **Endometriosis before inclusion** | 2,139 (16.4%) | 2,205 (16.9%) | <0.001 | 0.014 |

The consequences of this failed balancing are severe. The persistent imbalances indicate that the hysterectomy and non-hysterectomy groups are not comparable, and residual confounding is highly likely to be present. Therefore, the study's main finding of an HR of 1.42 cannot be confidently attributed to the effect of hysterectomy. This estimate may be biased upwards or downwards by the systematic differences between the groups that the PSM was intended to eliminate. This methodological failure is compounded by the authors' subsequent analytical decision. The footnote in Table 2 states, "Because propensity score matching was performed, hazard ratios were not adjusted for additional confounders". This statement reveals a critical misunderstanding. The decision to forego further adjustment in the Cox regression model is predicated on the assumption that PSM was successful. Since the matching demonstrably failed, this lack of further adjustment for the remaining imbalanced covariates is a major analytical error. Best practices in propensity score analysis dictate that if imbalances remain after matching, these should be controlled for in the final outcome model to mitigate bias.

The reasons for this failure warrant deeper consideration. While a large sample size (N=26,118) can render even clinically minor differences statistically significant (p < 0.05), the standardized mean difference (SMD) provides a more scale-invariant measure of balance. An SMD value below 0.1 is typically considered indicative of good balance. While some of the imbalanced variables have very small SMDs (e.g., 0.012 for uterine fibroids), the SMD for

Age at inclusion is 0.174, a value that clearly indicates a substantial and meaningful imbalance between the groups. The authors failed to critically evaluate both the p-values and the SMDs to assess the quality of their match.

More fundamentally, the failure of PSM may be a symptom of a deeper issue with the study design: profound confounding by indication. The hysterectomy group comprises women with significant benign gynecological diseases (e.g., fibroids, adenomyosis, abnormal bleeding) severe enough to warrant major surgery. The control group consists of women from the general population who attended a national health checkup. These are fundamentally dissimilar populations. The former is a clinical population defined by pathology, while the latter is a general population defined by a health-seeking behavior. It is highly probable that the covariates available within the NHIS administrative database are insufficient to capture the full spectrum of clinical and behavioral differences between these two groups. This questions the fundamental comparability of the chosen control group and, by extension, the internal validity of the entire study.

**Insufficient Statistical Power and Imprecision of the Effect Estimate**

The second major flaw that compromises the manuscript's conclusions is the study's lack of statistical power, which manifests as a profoundly imprecise primary effect estimate. The main result reported is an HR of 1.42 with a 95% CI of 0.79 to 2.56. While the point estimate suggests a 42% increased risk of ovarian cancer following hysterectomy, the confidence interval is exceptionally wide. It indicates that the true effect is statistically consistent with anything from a 21% risk reduction (HR 0.79) to a more than doubling of risk (a 156% increase, HR 2.56).

This imprecision is a direct and unavoidable consequence of the low number of outcome events. Ovarian cancer is a relatively rare disease. Despite a large cohort of 26,118 women and a median follow-up of 11.5 years, only 48 incident cases of ovarian cancer were identified (28 in the hysterectomy group and 20 in the non-hysterectomy group). This low event rate is the primary driver of the study's low statistical power. While the authors acknowledge this in their limitations section ("Fourth, there were few cases of incident ovarian cancer during the study period"), they fail to connect this limitation to the profound uncertainty it introduces into their main finding and the invalidity of their conclusion.

This leads to a critical error in scientific interpretation. The authors conclude, "Hysterectomy does not appear to significantly influence ovarian cancer risk" because the 95% CI crosses the null value of 1.0 (and thus the p-value is >0.05). This conclusion misapplies a core statistical principle: the absence of evidence is not evidence of absence. A non-significant p-value in an underpowered study does not prove the null hypothesis (i.e., that there is no true association). It merely indicates that the study lacked a sufficient sample of events to detect an association, even if one truly exists. The study's result is not a definitive null finding; it is an inconclusive one.

A more accurate and scientifically responsible conclusion, based on the presented data, would be: "Our study was underpowered to precisely estimate the effect of hysterectomy on ovarian cancer risk. The results are inconclusive and are statistically compatible with both a clinically meaningful increase in risk and a potential protective effect." By presenting their inconclusive result as a definitive null finding, the authors overstate the strength of their evidence and provide a misleading summary of their research.

This overstatement has direct and potentially harmful clinical implications. The recommendation that "Clinicians should not recommend hysterectomy for ovarian cancer risk reduction" is reasonable, as there is no clear evidence of a protective effect. However, the study's data, with an upper confidence limit of 2.56, cannot provide any reassurance to clinicians or patients that hysterectomy does not

*increase* the risk. A reader of this paper could wrongly infer that the procedure is neutral or safe with respect to ovarian cancer, a conclusion that the weak and imprecise data cannot support.

**Detailed Section-by-Section Appraisal**

**Title, Abstract, and Keywords**

The title, "Effect of hysterectomy on the risk of ovarian cancer: A South Korean national cohort study," is accurate and appropriate for the research question. The keywords (hysterectomy, ovarian cancer, cohort, Korea, salpingo-oophorectomy) are likewise relevant and well-chosen.

The abstract, however, is problematic. While it accurately summarizes the manuscript's content and numerical findings, it perpetuates the flawed interpretation of the main result. It presents the HR of 1.42 (95% CI 0.79-2.56) and the conclusion of no significant influence without acknowledging the profound statistical uncertainty and imprecision of this estimate. An abstract should convey not only the point estimate but also the degree of certainty around it. By omitting this context, the abstract presents a misleadingly definitive conclusion that is not supported by the weak underlying data.

**Introduction**

The introduction is well-written and effectively frames the research. It establishes hysterectomy as a common surgical procedure with known long-term health ramifications, particularly concerning ovarian function and premature menopause. The authors correctly identify a significant research gap, noting that most prior studies have been conducted in Western countries, which limits generalizability to Asian populations.

The stated hypothesis is biologically plausible and provides a strong rationale for the study. The authors hypothesize that hysterectomy, by reducing ovarian function and thus the frequency of ovulatory cycles, might *lower* the risk of ovarian cancer, consistent with the "incessant ovulation" theory. This well-reasoned hypothesis makes the study's final, albeit unreliable, result of a non-significant trend toward

*increased* risk (HR 1.42) particularly noteworthy and deserving of rigorous methodological scrutiny.

**Materials and Methods**

The choice of a retrospective cohort design using the comprehensive Korean NHIS database is an appropriate and powerful strategy for addressing the research question. Such databases allow for the assembly of large, population-based cohorts with extensive longitudinal follow-up data. However, the reliance on pre-existing administrative data carries inherent risks of information bias and unmeasured confounding, which are common challenges in such research.

The participant selection criteria are generally sound. The exclusion of women with a history of cancer or pre-existing benign ovarian diseases within one year of enrollment is a crucial and well-executed step to minimize selection bias and confounding by indication.

A notable strength of the methodology is the definition of the primary outcome. Defining incident ovarian cancer as requiring "three or more medical visits... with a diagnosis code (C56.xx)" is a robust strategy to increase the specificity and positive predictive value of case identification using administrative claims data. This approach helps to filter out provisional or rule-out diagnoses, a known limitation of claims-based research. While this method is a pragmatic proxy and does not replace histopathological confirmation, its validity for identifying true cases is likely high.

The primary weakness of this section lies in the description of the statistical analysis. While the authors state they used PSM and Cox proportional hazards models, they fail to provide crucial details about the PSM procedure. Key parameters such as the specific matching algorithm (e.g., nearest neighbor, optimal), the caliper width used to define a suitable match, and the ratio of matching are not specified. This lack of methodological detail, combined with the poor balancing results shown in Table 1, suggests a superficial or flawed application of a complex statistical technique.

**Results**

The numerical data presented in the text of the results section are consistent with the values reported in the accompanying tables and figures. However, the tables themselves contain significant errors and reveal the study's core methodological failure.

As detailed extensively in Section II.A, Table 1 is the primary evidence of the failed PSM. The presence of highly significant p-values and, in some cases, large SMDs for key confounders after matching is a critical flaw that invalidates the assumption of comparability between the study groups.

Table 2 contains a clear typographical or data presentation error. For the subgroup "Hysterectomy with adnexal surgery," the value for "Ovarian cancer cases per 100,000 person-years" is listed as "310,194 /(29)". This entry is nonsensical and must be corrected. Furthermore, the wide confidence intervals for all subgroups reported in Tables 2 and 3 consistently underscore the study's low statistical power and the unreliability of the point estimates.

Figure 2, the Kaplan-Meier plot, visually confirms the results of the Cox model, showing no statistically significant separation between the survival curves for the two groups (stratified log-rank test p-value = 0.2).

**Discussion**

The discussion section fails to grapple with the study's most significant shortcomings. The authors proceed with their interpretation as if the HR of 1.42 (95% CI 0.79-2.56) is a robust and unbiased estimate, which it is not. The entire interpretation is built on a methodologically flawed foundation.

The authors provide a competent review of prior literature, including meta-analyses that largely found a protective effect of hysterectomy on ovarian cancer risk. They offer two plausible explanations for the discrepancy between their findings and this prior work: 1) a shorter follow-up period in their study, and 2) potential confounding by the use of menopausal hormone therapy (MHT) after hysterectomy. While these are valid points for consideration, the authors miss the most obvious and critical explanation for the discrepancy: their own study's result may be spurious due to uncontrolled confounding arising from the failed PSM. The difference may not be a true biological or temporal phenomenon but rather an artifact of poor study execution.

The acknowledged strengths and limitations are mostly appropriate. However, the most critical limitation—the failure of the propensity score matching to achieve covariate balance—is conspicuously absent from their list. This is a major omission that suggests a lack of critical self-appraisal of their own methodology. Similarly, while they mention the low number of cases as a limitation, they significantly downplay its impact on the statistical power and the profound uncertainty of their conclusion.

**Conclusions**

As argued in Section II.B, the study's main conclusion—"Hysterectomy was not associated with a significant change in ovarian cancer risk among Korean women"—is an overstatement of the evidence. The data are inconclusive due to low statistical power and the wide confidence interval. The study failed to provide a clear answer, and the conclusion must be revised to reflect this fundamental uncertainty. The clinical recommendation is reasonable but should be framed more cautiously, explicitly stating that while no protective effect was found, a clinically meaningful increased risk could not be ruled out by their data.

**Minor Points and Formal Aspects**

There are several formal aspects of the manuscript that require correction. Most notably, the data availability statement is contradictory. The submission form indicates, "Yes - all data are fully available without restriction". However, the acknowledgments section correctly states, "Data sharing: Raw data cannot be provided per National Health Insurance Service (NHIS)'s privacy policy". This must be rectified to accurately reflect that the data are owned by a third party (the NHIS) and are available only to researchers who meet the criteria for access, not without restriction.

Apart from this, the manuscript is generally well-written, with a clear logical flow and structure. The ethics and funding statements are complete, consistent, and appropriate for this type of research.

**Overall Recommendation and Concluding Remarks**

This study addresses an important and unresolved clinical question in a highly relevant but understudied population. The use of a national cohort database is a significant strength. However, the execution of the study is compromised by fundamental methodological flaws and a critical lack of statistical power. The failure of the propensity score matching to balance key covariates leaves the results open to significant confounding bias, and the low number of outcome events renders the main finding inconclusive.

The conclusions drawn by the authors are not supported by the weak and unreliable evidence presented. To be considered for publication, the manuscript would require, at a minimum, the following substantial revisions:

1. **Address the Failed Propensity Score Matching:** The authors must undertake a complete re-analysis. This should involve attempting more sophisticated matching algorithms (e.g., optimal matching, caliper restrictions) or employing alternative methods for confounding control, such as inverse probability of treatment weighting (IPTW). If imbalances persist, multivariable adjustment for all imbalanced covariates must be performed in the final Cox regression model.
2. **Present Validated Results:** All results must be recalculated and presented based on a statistically valid approach that demonstrably controls for confounding.
3. **Revise Interpretation and Conclusions:** The discussion and conclusions must be thoroughly rewritten to honestly and transparently reflect the limitations of the data, particularly the low statistical power and the resulting imprecision of the effect estimate. The conclusion must be softened from a definitive statement of "no association" to a more appropriate acknowledgment of inconclusive findings.
4. **Correct Factual Errors:** The typographical error in Table 2 and the contradictory data availability statement must be corrected.

Given the current state of the manuscript, the recommendation is **Rejection**. The flaws are not minor issues that can be resolved with simple textual edits; they are fundamental to the study's internal validity and the reliability of its conclusions. A major re-analysis and a complete rewriting of the manuscript's interpretation would be necessary to make this work potentially acceptable for publication.
