# Supplementary material for: Effect of hysterectomy on the risk of ovarian cancer: A South Korean national cohort study
Source: PLoS One. 2026 May 6;21(5):e0348127. doi: 10.1371/journal.pone.0348127 (PMC13148655; doi:10.1371/journal.pone.0348127)
Supplement: Attachment — Submitted filename: 2025-11-01_Author Response Letter_v1.docx [file pone.0348127.s005.docx]

**Reviewer comment**

**Response to Reviewer #1 Comments**

We thank the reviewer for highlighting both the strengths and methodological challenges of our cohort study. Below, we respond directly and assertively to each major point with supporting evidence from our data and current analytic standards.

**1. Propensity Score Matching (PSM), SMD, and Covariate Balance**

**Reviewer comment:**

PSM yielded residual imbalance in key covariates including age, with SMD reported as 0.174. This is interpreted to indicate the matching failed.

**Response:**

Contrary to the assertion that an SMD 0.174 signifies inadequate balance, the established epidemiological and statistical consensus defines an SMD < 0.2 as reflecting acceptable balance between matched cohorts[1, 2]. In our study, all reported SMDs after matching, including the cited value for age, were below this widely recognized threshold, indicating adequate matching and effective minimization of confounding. As detailed in Table 1 and supplementary analyses, residual imbalances were minor and are unlikely to materially influence the primary outcome.

Furthermore, sensitivity analyses in subjects with zero comorbidity (CCI = 0) confirm the robustness of our inference, yielding consistent hazard ratios for ovarian cancer. We therefore strongly contend that our matching procedure meets accepted methodological standards and that our results can be interpreted with scientific confidence.

**2. Statistical Power (Event Number) and Conclusion Validity**

**Reviewer comment:**

The low number of ovarian cancer cases leads to wide confidence intervals; the conclusion should be “inconclusive” rather than “no effect.”

**Response:**

We acknowledge the limited number of events and the wide confidence intervals in our analysis. However, these event rates reflect the real-world rarity of ovarian cancer, particularly in Asian populations over a median follow-up of 11.5 years. Our cohort of over 26,000 women is among the largest Asian hysterectomy samples published to date. Importantly, the direction of the hazard ratio remained consistent across sensitivity and subgroup analyses. Nevertheless, in response to this criticism, we have explicitly revised our conclusion language to acknowledge the limitations in statistical power and now state that the results are “not statistically significant and inconclusive” regarding an association.

Revised manuscript (Discussion part): “In conclusion, hysterectomy was not statistically significantly associated with ovarian cancer risk in Korean women. However, due to limited events and wide confidence intervals, these findings are inconclusive. Further research is needed before clinical recommendations regarding hysterectomy for ovarian cancer risk reduction can be confidently made.”

**3. Analytical Interpretation (Absence of Evidence ≠ Evidence of Absence)**

**Reviewer comment:**

Nonsignificant p-values or confidence intervals should not be over-interpreted as absence of effect.

**Response:**

We agree with this methodological principle. Throughout the revised manuscript, all relevant sections now clarify that while our findings are not statistically significant, they do not demonstrate evidence of absence; the true relationship remains uncertain due to sample size and event limitation.

Revised manuscript (Abstract part): “Hysterectomy does not appear to significantly alter ovarian cancer risk among Korean women; however, the limited number of ovarian cancer cases and wide confidence intervals result in inconclusive findings. Further studies are needed before establishing clinical recommendations.”

Revised manuscript (Discussion part): “In conclusion, hysterectomy was not statistically significantly associated with ovarian cancer risk in Korean women. However, due to limited events and wide confidence intervals, these findings are inconclusive. Further research is needed before clinical recommendations regarding hysterectomy for ovarian cancer risk reduction can be confidently made.”

**4. Minor Issues (Tables, Data Sharing Policy)**

**Reviewer comment:**

Typographical errors in tables; confusion in the statement about data sharing.

**Response:**

All typographical errors in the revised tables have been corrected.

Regarding data sharing, the National Health Insurance Service of Korea enforces strict privacy regulations that prohibit the disclosure of raw data. This restriction has now been clearly stated in the revised manuscript, in full compliance with NHIS policy. While the methods and acknowledgments sections of the manuscript correctly indicate that data sharing is not permitted, the submission form previously stated otherwise, which may have caused confusion. To resolve this inconsistency, we have removed the phrase “all data are fully available without restriction” from the submission form.

**5. Feasibility of Re-analysis**

**Reviewer comment:**

Further analyses (enhanced matching, IPTW, or covariate adjustments) are requested.

**Response:**

We recognize the value of such analyses. However, the official analytic period for the dataset has concluded and the NHIS system now prohibits all further access, as per Korean government guidelines for privacy and IT security. This practical and legal constraint is explicitly stated in the revised manuscript.

**Response to Reviewer #2 Comments**

We thank Reviewer #2 for their careful reading, constructive feedback, and positive remarks about the significance and potential of our study. We respond to each point individually below, providing a robust and evidence-based defense for our methods and addressing all editorial and methodological comments.

**1. Introduction: Punctuation and In-text Citation Style**

**Reviewer comment:**

Punctuation should follow, not precede, in-text citations. PLOS ONE requires square brackets for citations.

**Response:**

We appreciate the reviewer’s attention to editorial detail. We have systematically revised the manuscript so that all in-text citations now use square brackets, with punctuation placed after the reference, consistent with PLOS ONE guidelines. This improves compliance and readability throughout the introduction and main text.

**2. Materials and Methods: PSM, SMD, and Anderson-Darling Test**

**Reviewer comment:**

The necessity of evaluating match quality is highlighted. Reviewer requests results of the Anderson–Darling normality test and questions the representativeness of the sample.

**Response:**

We agree on the importance of formally evaluating the distributional properties of covariates after matching. We performed the Anderson–Darling normality test for all continuous covariates in our matched samples, which uniformly resulted in p-values < 0.001, indicating violations of normality assumptions. In accordance with these results, we applied the Wilcoxon signed-rank test for between-group comparisons of continuous variables rather than parametric approaches, ensuring statistical validity. These procedures and testing outcomes are now explicitly described in the revised Methods and Results sections.

Revised manuscript (Methods part): “After propensity score matching, categorical variables were analyzed with the Cochran–Mantel–Haenszel test. Continuous variables were compared using the paired t-test and, given that the Anderson–Darling normality test indicated significant departures from normality for all variables (all p-values < 0.001), we relied primarily on the nonparametric Wilcoxon signed-rank test to ensure statistical validity. Covariate balance was evaluated using standardized mean differences.”

Revised manuscript (Results part): “The Anderson–Darling normality test applied to continuous covariates in the matched sample uniformly yielded p-values < 0.001, indicating non-normal distributions; therefore, we used the Wilcoxon signed-rank test for group comparisons. Covariate balance was confirmed, with all standardized mean differences (SMDs) below 0.2 (Table 1).”

Regarding representativeness, our cohort was drawn from the comprehensive Korean National Health Insurance Service (NHIS) database, which covers virtually the entire South Korean population. Thus, our sample is highly representative and generalizable to the national demographic.

**3. Discussion: Editorial Comments**

**3.1 Western vs. Asian Studies Wording**

**Reviewer comment:**

Suggested improvements to the phrasing distinguishing Western and Asian research.

**Response:**

We have adopted the reviewer’s proposed revision to enhance clarity.

Revised manuscript (Discussion part): “Finally, most studies on this topic have been conducted in Western countries, including the USA, Canada, Denmark, Sweden, the Netherlands, and Finland. In contrast, our study pioneered research in Asian populations.”

**3.2 Editing of Strengths and Limitations**

**Reviewer comment:**

Recommends editing for coherence and readability.

**Response:**

We accept this valuable suggestion. The strengths and limitations sections have been thoroughly reviewed and edited to maximize clarity and logical flow, as detailed in the revised Discussion.

Revised manuscript (Discussion part): “This study has several notable strengths. First, it utilizes a large, nationally representative cohort drawn from the comprehensive Korean National Health Insurance Service database, ensuring broad generalizability to South Korean women. The dataset’s high quality and longitudinal design, featuring a substantial sample size and extended follow-up, enhance statistical power and enable robust outcome assessment. Confounding factors were rigorously controlled through strict inclusion and exclusion criteria and detailed propensity score matching across a wide range of demographic and clinical variables, with balance confirmed by standardized mean differences (all SMDs < 0.2). Additionally, subgroup and sensitivity analyses further strengthened the internal validity of our findings. Finally, most studies on this topic have been conducted in Western countries, including the USA, Canada, Denmark, Sweden, the Netherlands, and Finland. In contrast, our study pioneered research in Asian populations.

Nonetheless, some inherent limitations merit acknowledgment. We lacked information on individual genetic risk (e.g., BRCA mutations) and family history of ovarian cancer, restricting exploration of high-risk populations. As a retrospective cohort analysis, the potential for unmeasured confounding remains, despite comprehensive covariate adjustment. The limited number of ovarian cancer events narrows precision and statistical power for subgroup analyses, necessitating cautious interpretation. Lastly, our cohort consisted exclusively of Korean women, which—while relevant for Asian populations—may limit direct extrapolation of findings to other ethnic groups.”

**3.3 Repetition in Discussion and Conclusion**

**Reviewer comment:**

The end of the Discussion repeats concluding statements, which are then also stated in the Conclusion; suggests omitting redundancy.

**Response:**

To avoid unnecessary repetition, the conclusions with overly broad titles have been removed and are now briefly summarized at the end of the Discussion section. In accordance with the reviewers' editorial guidelines, the main conclusions are confined to the Discussion section.

Revised manuscript (Discussion part): “In conclusion, hysterectomy was not statistically significantly associated with ovarian cancer risk in Korean women. However, due to limited events and wide confidence intervals, these findings are inconclusive. Further research is needed before clinical recommendations regarding hysterectomy for ovarian cancer risk reduction can be confidently made.”

1.	Stuart, E.A., *Matching methods for causal inference: A review and a look forward.* Stat Sci, 2010. **25**(1): p. 1-21.

2.	Austin, P.C., *Balance diagnostics for comparing the distribution of baseline covariates between treatment groups in propensity-score matched samples.* Stat Med, 2009. **28**(25): p. 3083-107.
