# Supplementary material for: Effect of hysterectomy on the risk of ovarian cancer: A South Korean national cohort study
Source: PLoS One. 2026 May 6;21(5):e0348127. doi: 10.1371/journal.pone.0348127 (PMC13148655; doi:10.1371/journal.pone.0348127)
Supplement: Attachment — Submitted filename: 2026-01-14 PLoS One revision_v2.docx [file pone.0348127.s006.docx]

**Reviewer #1:**

We thank for their careful reading, constructive feedback, and positive remarks about the significance and potential of our study. We respond to each point individually below, providing a robust and evidence-based defense for our methods and addressing all editorial and methodological comments.

**1. Abstract**
Mistake/Weakness: The hazard ratio in the Abstract (HR 1.42) differs slightly from the one reported in the main text (Table 2, HR 1.421). While minor, consistency is key. The rounded value should be the same (1.42).

**Response:** We thank the reviewer for pointing out the small discrepancy in the hazard ratio reported in the Abstract and Table 2. In the revised manuscript, we have ensured consistency by rounding the HR to two decimal places throughout the text, including the Abstract (HR 1.42, 95% CI 0.79–2.56), in line with the value presented in Table 2.

Weakness: The conclusion states, "Hysterectomy does not appear to significantly alter ovarian cancer risk..." This phrasing, while technically true based on the p-value, is misleading. A more accurate summary would be, "This study found no statistically significant association, but the wide confidence intervals preclude a definitive conclusion on whether hysterectomy increases, decreases, or has no effect on risk." The authors do mention "inconclusive findings" in the next sentence, but the primary conclusion is softly stated as "no effect."

**Response**: We agree with the reviewer that our Abstract conclusion could be interpreted as implying a ‘no effect’ conclusion. To avoid this, we have revised the Abstract conclusion to explicitly emphasize the lack of statistical significance and the imprecision of our estimates. The revised sentence now reads (lines 45~46): “This study found no statistically significant association between hysterectomy and ovarian cancer risk, but the wide confidence intervals and limited number of events indicate that the findings remain inconclusive.”

**2. Introduction**
Weakness: The biological rationale is somewhat one-sided. The introduction focuses heavily on the hypothesis that hysterectomy might reduce ovarian cancer risk (via reduced ovarian function/ovulation). However, it largely ignores plausible mechanisms for a null or even increased risk (e.g., compensatory estrogen-only MHT use, as they later mention in the discussion, or the fact that simple hysterectomy leaves the primary site of origin for high-grade serous cancer—the fallopian tubes—in place). This sets up a expectation of a protective effect that the study then fails to find.

**Response:** We appreciate the reviewer’s insightful comment on the biological rationale in the Introduction. We agree that the text previously emphasized the hypothesis of a protective effect via reduced ovarian function and ovulation, while underrepresenting mechanisms that could lead to a null or even increased risk. In the revised Introduction, we have added a paragraph acknowledging that simple hysterectomy leaves the fallopian tubes in place, which are now considered a major site of origin for high-grade serous ovarian cancer, and that hysterectomy induced earlier menopause may lead to prolonged use of estrogen-only menopausal hormone therapy, which has been linked to increased ovarian cancer risk. We also now explicitly state that, because of these competing mechanisms, the net effect of hysterectomy on ovarian cancer risk is biologically uncertain and could plausibly range from risk reduction to no effect or risk increase. The revised sentence now reads (Lines 78~89): “Conversely, several biological mechanisms support the possibility of a null or even increased risk after simple hysterectomy. Simple hysterectomy leaves the fallopian tubes in situ, and accumulating pathologic and epidemiologic evidence indicates that many high-grade serous ovarian, fallopian tube, and primary peritoneal carcinomas arise from the distal fallopian tube epithelium rather than the ovarian surface itself. Therefore, unlike salpingectomy or salpingo-oophorectomy, simple hysterectomy does not remove the presumed primary site of origin for these tumors. In addition, hysterectomy-induced earlier menopause may lead to prolonged use of estrogen-only menopausal hormone therapy (MHT) in women without a uterus, and estrogen-only MHT has been associated with an increased risk of ovarian cancer in observational studies. These competing mechanisms suggest that the net effect of hysterectomy on ovarian cancer risk is biologically uncertain and could plausibly range from risk reduction to no effect or even risk increase.”, (Lines 95~98): “Given the high prevalence of hysterectomy and the emerging recognition of fallopian tube–origin high-grade serous carcinogenesis, clarifying the long-term ovarian cancer risk after simple hysterectomy in diverse racial and healthcare contexts is of considerable clinical importance.”, (Lines 101~103): “Rather than presuming a strictly protective effect, our study was designed to empirically assess whether hysterectomy is associated with a decreased, unchanged, or increased risk of ovarian cancer in this population.”

**3. Methods**
Study Design & Database: Well-described and appropriate.

**Participant Selection**

Weakness: The "non-hysterectomy" group is defined as women who underwent a health checkup. This introduces a "healthy user" bias. Women who attend regular checkups may be systematically different from those who do not (e.g., more health-conscious, higher socioeconomic status), which could independently influence their ovarian cancer risk.

**Response:** We appreciate the reviewer’s thoughtful comment regarding potential “healthy user” bias arising from the use of health checkup attendees as the non-hysterectomy comparison group. We agree that women who attend regular checkups may differ from non-attendees in terms of health behaviors and socioeconomic characteristics, which could influence ovarian cancer risk. In our study, we attempted to mitigate this concern by applying rigorous propensity score matching on a wide range of variables, including age, socioeconomic status, smoking, alcohol consumption, physical activity, parity, menopausal status, comorbidities, and gynecologic conditions, all of which were measured within the same national health screening program. This approach ensured that both groups were drawn from the same underlying source population (NHIS beneficiaries undergoing health examinations) and were well balanced on observed characteristics. Nevertheless, we fully acknowledge that residual confounding by unmeasured factors related to health-seeking behavior may remain. To address this, we have explicitly added “potential healthy user bias due to the use of health checkup attendees as the comparison group” as a limitation in the Discussion and clarified that this bias may have influenced the observed association. The revised sentence now reads (lines 367-370): “Although we applied propensity score matching using socioeconomic and lifestyle variables from the same screening program, residual confounding related to health-seeking behavior may still remain.”

**Outcomes**

Weakness: Defining ovarian cancer as "three or more visits... with a diagnosis code" is a good attempt to increase specificity and reduce false positives. However, the validity of this algorithm is not tested or referenced. Without linkage to a cancer registry to confirm the diagnosis, some misclassification is possible.

**Response:** We thank the reviewer for the thoughtful comment regarding the validity of our claims-based ovarian cancer definition.

While we did not link our cohort directly to the Korean Central Cancer Registry (KCCR) in this study, we attempted to increase the specificity of outcome ascertainment by defining incident ovarian cancer as three or more healthcare visits with an ICD-10 diagnosis code C56.xx (primary or secondary diagnosis).

Importantly, in the Korean healthcare system, malignant neoplasms coded as “C-codes” are closely related to the Rare and Intractable Disease (RID) registration / expanded benefit system, which provides substantial copayment reductions and therefore requires documentation and review for registration. This administrative structure generally discourages casual or inaccurate assignment of C-codes compared with ordinary diagnosis codes.

In addition, prior validation work using KCCR–NHIS linked data has shown that operational definitions based on NHIS claims (particularly those using RID registration claims) achieve high sensitivity (generally >90%, varying by cancer type) and that diagnosis timing based on claims is often close to registry diagnosis dates, supporting the feasibility of cancer outcome definitions using NHIS data[1].

We have now cited this validation literature and expanded our discussion of potential outcome misclassification as a limitation.

(Discussion-Limitation lines 357-366) In addition, outcome misclassification is possible because ovarian cancer was ascertained from claims data and we did not link our analytic dataset directly to the Korean Central Cancer Registry (KCCR). However, a Korean validation study using a KCCR–NHIS linked database reported that operational definitions based on NHIS claims showed high sensitivity (>90%) for major cancers when using either primary diagnosis codes or RID registration claims, and that >80% of patients had a <31-day difference between the registry diagnosis date and the RID-based claims definition[1]. That study also suggested that the accuracy of RID-based definitions improved after the mid-2000s as the RID program became established, which should be considered when interpreting claims-based case definitions across earlier calendar years.

**Statistics**

Major Weakness - Power Calculation: There is no mention of a pre-study power or sample size calculation. This is a critical omission. Given the low incidence of ovarian cancer, the authors should have estimated the number of events needed to detect a clinically meaningful hazard ratio (e.g., HR=0.7 or 1.3) before conducting the study. The fact that they ended up with only 48 total events is the fundamental limitation of this paper, and the lack of a power calculation makes it seem like this was an unforeseen problem rather than a known limitation of the study design from the outset.

**Response:** We agree that the manuscript should state whether a pre-study power/sample size calculation was performed and should explicitly contextualize the implications of the low ovarian cancer incidence in this dataset.

Because this was a retrospective cohort study using the fixed Korean NHIS database (2002–2020), the available sample size was determined by eligibility criteria and the NHIS analytical-server sampling constraints, rather than by a prospectively planned recruitment target.

After propensity score matching, 13,059 women were included in each group, and the analysis ultimately observed only 48 ovarian cancer events, which led to wide confidence intervals around the main estimate (HR 1.42, 95% CI 0.79–2.56).

To address the reviewer’s request, we added a power/event calculation to quantify this limitation. Using Schoenfeld’s method for Cox models (two-sided α=0.05), approximately 247 events would be required for 80% power to detect HR=0.7, and approximately 456 events would be required for 80% power to detect HR=1.3.

With only 48 total events, the estimated power is ~23% for HR=0.7 and ~15% for HR=1.3, meaning the study is only adequately powered for very large effects (approximately HR <0.45 or >2.24 for 80% power).

Accordingly, we revised the manuscript to make clear that limited power was an inherent design constraint given the rarity of ovarian cancer, rather than an unforeseen issue.

(Methods lines 138-140): “Because this was a retrospective database study with a fixed study population determined by NHIS availability and eligibility criteria, we did not perform a pre-study sample size calculation.”

(Discussion/Limitations lines 349-352): “Using Schoenfeld’s method for Cox models (two-sided α=0.05), ~247 events would be required for 80% power to detect HR=0.7 and ~456 events for HR=1.3; with only 48 events, power is ~23% and ~15%, respectively, and the study is only powered for very large effects (approximately HR <0.45 or >2.24).”

Weakness (Addressed but noteworthy): The use of SMD < 0.2 as a balance threshold is standard, but the reviewer correctly pointed out that an SMD of 0.174 for age is not ideal. The authors' defense is statistically correct but clinically, a residual imbalance in the most important confounder (age) is not trivial.

**Response:** Thank you for this important comment. We agree that, although SMD <0.2 is a commonly used balance threshold, the residual imbalance in age (SMD 0.174) is not ideal because age is the most important confounder for ovarian cancer risk.

To address this, we conducted an additional multivariable Cox proportional hazards analysis within the propensity score–matched cohort, adjusting for all baseline covariates used in the propensity score model (including age), to mitigate potential residual confounding.

After this additional adjustment, the association between hysterectomy and ovarian cancer remained not statistically significant: hysterectomy (with/without adnexal surgery) HR 1.22 (95% CI 0.56–2.68; P=0.621), hysterectomy without adnexal surgery HR 1.16 (0.49–2.73; P=0.732), and hysterectomy with adnexal surgery HR 1.66 (0.17–15.79; P=0.659).

These findings support that the main conclusion is robust even when accounting more strictly for residual imbalance in age and other covariates after matching.

(Section 2.5 Statistics; lines 179-182): “To address potential residual covariate imbalance after propensity score matching (particularly for age), we additionally fitted a multivariable Cox proportional hazards model in the matched cohort adjusting for all baseline covariates included in the propensity score model.”

(Section 3 Results; lines 231-237): “Because age remained the largest residual imbalance after matching (SMD 0.17), we performed a multivariable Cox regression in the matched cohort additionally adjusting for all baseline covariates (including age). The adjusted hazard ratios remained not statistically significant: hysterectomy (with/without adnexal surgery) HR 1.22 (95% CI 0.56–2.68; P=0.621), hysterectomy without adnexal surgery HR 1.16 (0.49–2.73; P=0.732), and hysterectomy with adnexal surgery HR 1.66 (0.17–15.79; P=0.659).”

**4. Results**
Weakness: The presentation of the main result is statistically correct but epidemiologically problematic. Stating a Hazard Ratio of 1.421 (suggesting a 42% increased risk) with a confidence interval of 0.79–2.56 is not evidence of "no effect." It is evidence of extreme uncertainty. The data are compatible with everything from a 21% risk reduction to a 156% risk increase. The Kaplan-Meier curve visually supports this high degree of uncertainty, with the lines virtually overlapping.

**Response:** Thank you for this insightful epidemiologic clarification. The main estimate (HR 1.42, 95% CI 0.79–2.56) is not evidence of “no effect”; instead, it reflects substantial imprecision due to the small number of ovarian cancer events.

We agree that the confidence interval indicates the data are compatible with a range of effects—from approximately a 21% risk reduction (0.79) to a 156% risk increase (2.56)—and therefore the appropriate interpretation is that the findings are inconclusive rather than null.

We also agree that the Kaplan–Meier curves showing near-overlap (stratified log-rank p=0.2) are consistent with limited separation and high uncertainty, not proof of equivalence or absence of effect.

■Main text modifications

■Abstract

(lines 40) Revised wording to avoid implying “no effect” and to explicitly emphasize imprecision.

Original: “Hysterectomy was not associated with a statistically significant change in ovarian cancer risk (HR 1.42, 95% CI 0.79–2.56).”

Revised (lines 40-42): “Hysterectomy was associated with an imprecise estimate of ovarian cancer risk (HR 1.42, 95% CI 0.79–2.56), compatible with both a clinically meaningful decrease and increase in risk; therefore, the findings are inconclusive.”

■Results

(lines 216; immediately after reporting HR 1.42, 95% CI 0.79–2.56) Added a sentence explicitly describing the uncertainty represented by the CI rather than treating non-significance as “no association.”

Added text (lines 216-218): “The wide confidence interval indicates substantial uncertainty, and the data remain compatible with both risk reduction and risk increase.”

(lines 210) The manuscript already reports the Kaplan–Meier analysis and the stratified log-rank p-value (p=0.2); we added a brief interpretive clarification that overlap reflects limited information rather than equivalence.

Added text (lines 211-213): “The near-overlapping curves should be interpreted as reflecting limited event information and imprecision rather than evidence of no effect.”

■ Conclusion

(lines 378) Revised conclusion to avoid “not associated” phrasing.

Original: “Hysterectomy was not statistically significantly associated with ovarian cancer risk…”

Revised (lines 378-380: “Hysterectomy was associated with an imprecise, statistically non-significant estimate of ovarian cancer risk; due to limited events and wide confidence intervals, the findings are inconclusive.”

Major Weakness - Subgroup Analyses: The subgroup analyses are severely underpowered and should not have been performed, or their results should be dismissed. For example, the subgroup "Hysterectomy with adnexal surgery" has only 3 events, yielding a nonsensical HR of 1.5 with a CI of 0.251–8.977. Presenting these results in a table gives them a false sense of validity. They are uninterpretable.

**Response:** Thank you for this important comment. We agree that the subgroup analyses are severely underpowered, and that the subgroup “hysterectomy with adnexal surgery” (3 ovarian cancer events) yields an extremely unstable estimate (HR 1.50 with a very wide CI 0.25–8.98), which is not interpretable for epidemiologic inference. We also agree that placing such sparse-event subgroup estimates in a main Results table may give a false sense of precision/validity despite the extreme imprecision. Accordingly, we revised the manuscript to de-emphasize these subgroup analyses and to prevent over-interpretation by readers.

■Results (remove/de-emphasize subgroup HRs)

(lines 218; Results text describing “hysterectomy with/without adnexal surgery” subgroups) We removed the detailed reporting of subgroup HRs from the main Results narrative to avoid implying interpretability.

Original text (summarized): The Results reported separate HRs for hysterectomy without adnexal surgery and hysterectomy with adnexal surgery.

Revised text (lines 218-220): “Subgroup analyses were exploratory and were not used for inference because of sparse events in some strata (e.g., adnexal surgery subgroup). Therefore, only the primary matched analysis is emphasized in the main Results.”

■Tables (avoid false validity)

(Table 2) We removed the “hysterectomy with adnexal surgery” subgroup row from the main table (or alternatively moved all subgroup rows out of Table 2), because the estimate is driven by only 3 events and is not interpretable.

(Table 3) We moved the age-stratified subgroup table out of the main manuscript (to Supplementary Table 2) and explicitly labeled it “exploratory” due to limited events per stratum.

■Discussion (explicit limitation)

(Limitations; lines 355) We added a sentence clarifying that subgroup analyses with sparse events are unstable and should not be interpreted as evidence for effect modification.

Added text (lines 355-357): “Exploratory subgroup estimates were unstable due to sparse outcome events in some strata (e.g., adnexal surgery subgroup), and therefore were not emphasized for inference.”

**5. Discussion**
Strengths: The discussion does a good job of comparing findings to previous meta-analyses and proposing plausible explanations for the null result (shorter follow-up, MHT use).

Weaknesses
Over-interpretation: The opening sentence, "this study found that hysterectomy is not significantly associated with ovarian cancer," is an overstatement. A more accurate phrasing would be "this study did not find a statistically significant association."

**Response:** We appreciate the reviewer’s suggestion regarding the wording of our main conclusion. We agree that the previous phrasing could be interpreted as implying evidence of no association, whereas our data actually indicate considerable uncertainty. In line with the reviewer’s recommendation, we have revised the opening sentence of the Discussion to state that (line 244-247) “Using national population-based cohort data, this study did not find a statistically significant association between hysterectomy and ovarian cancer risk among middle-aged and older women in Korea (HR 1.42, 95% CI 0.79–2.556), but the wide confidence intervals indicate considerable uncertainty regarding the true magnitude and even the direction of the association” and have further emphasized the wide confidence intervals and limited power.

Ignoring the Point Estimate: The discussion focuses on the lack of significance and the wide CIs but does not adequately address the point estimate of HR=1.42. If this is a true effect, it would be clinically important. The possibility of an increased risk should be discussed more thoroughly, not just the failure to find a decreased risk.

**Response:** We thank the reviewer for highlighting the importance of the point estimate. We agree that an HR of 1.42, if true, would represent a clinically meaningful increase in ovarian cancer risk. In the revised Discussion, we now explicitly comment on the point estimate and its clinical implications, while at the same time stressing that the wide confidence interval and limited number of events preclude firm conclusions. We also acknowledge that our study cannot rule out the possibility of a modest to moderate risk increase associated with hysterectomy. The revised sentence now reads (lines 248-254): “Although the association did not reach statistical significance, the point estimate (HR 1.42) suggests a possible increase in ovarian cancer risk after hysterectomy. If this estimate reflected the true effect, it would be clinically important. However, the wide 95% confidence interval (0.79–2.56) indicates that our data are also compatible with a modest risk reduction and with a substantially larger risk increase. Given the limited number of ovarian cancer events in our cohort, our study cannot reliably distinguish among these possibilities and therefore cannot exclude a clinically meaningful increase in risk.”

Limitations Section: The limitation regarding the "limited number of ovarian cancer events" is correctly identified as the primary weakness. However, it should be stated more forcefully: "The study was severely underpowered to detect anything but a very large effect size, which fundamentally limits the conclusiveness of our findings."

**Response:** We agree with the reviewer that the limitation regarding statistical power should be articulated more forcefully. In the revised Limitations section, we now explicitly state that the study was severely underpowered to detect anything but very large effect sizes, which fundamentally constrains the interpretability and conclusiveness of our findings. We believe this revision better reflects the true strength of the available evidence. The revised sentence now reads (lines 347-349): “The most important limitation of this study is the limited number of ovarian cancer events, which resulted in wide confidence intervals and low statistical power.” (lines 352-355): “In particular, the study was severely underpowered to detect anything but a very large effect size, which fundamentally limits the conclusiveness of our findings and means that modest increases or decreases in risk cannot be ruled out.”

**Reviewer #2: (No Response)**

**Reviewer #3:**

This study addresses an important gap in gynecologic oncology literature regarding the long-term association between hysterectomy and ovarian cancer risk, specifically within the Asian population (South Korea). Utilizing the NHIS database provides a substantial sample size and minimizes recall bias. However, there are significant concerns regarding the definition of exposure groups, statistical power, and the interpretation of the "non-significant" findings which paradoxically lean towards an increased risk (HR > 1.0), contradictory to the protective mechanisms extensively discussed.

**Response:** We thank the reviewer for highlighting both the strengths and methodological challenges of our cohort study. Below, we respond directly and assertively to each major point with supporting evidence from our data and current analytic standards.

**1. Clarification of Exposure Definitions and "Adnexal Surgery" (Crucial)**

Observation: In the Abstract (Line 29-30), the objective mentions "simple hysterectomy (with or without bilateral salpingo-oophorectomy, BSO)". However, "Simple Hysterectomy" medically implies removal of the uterus only. Including BSO in the definition of "Simple Hysterectomy" is terminologically incorrect and confusing.

**Response:** We sincerely thank for identifying this important source of terminological confusion. We modified “simple hysterectomy” to “hysterectomy”.

Critical Issue: In the Discussion (Lines 216-217), the author reports: "hysterectomy with adnexal surgery (HR 1.5, 95% CI 0.251–8.977)."

If "adnexal surgery" implies Bilateral Salpingo-Oophorectomy (BSO), it is biologically implausible for the risk of ovarian cancer to increase (HR 1.5), unless these are cases of Primary Peritoneal Carcinoma (PPC) or Ovarian Remnant Syndrome.

If the ovaries were removed, how is the incidence defined?

Recommendation: The authors must rigorously define "adnexal surgery." Does it mean unilateral oophorectomy? Salpingectomy only? Or BSO? If it is BSO, the authors must explain the histology of the cancers found (e.g., are they actually peritoneal cancers miscoded as C56?).

**Response:** Thank you for raising this critical point. In the previous version, we reported an exploratory subgroup estimate for “hysterectomy with adnexal surgery” (HR 1.50, 95% CI 0.25–8.98), but this subgroup had only 3 ovarian cancer events and the estimate was therefore unstable and not interpretable. In response to the reviewer’s concerns about both sparse events and biologic plausibility (particularly if adnexal surgery included BSO), we removed the adnexal-surgery subgroup analysis from the main Results and deleted the corresponding sentence in the Discussion.

Separately, we agree that the term “adnexal surgery” must be explicitly defined in the manuscript, and we have added a clear operational definition based on NHIS procedure codes, specifying whether it refers to salpingectomy and/or oophorectomy (and, when identifiable, laterality).

■Methods (define “adnexal surgery”)

(Section 2.4 Confounding Variables; line 154) The manuscript currently states that “hysterectomy or adnexal surgery (recorded using surgery codes)” were considered.

Revised text (lines 159-160): “Concomitant adnexal surgery was defined using NHIS procedure codes indicating salpingectomy and/or oophorectomy performed at the time of hysterectomy.”

2. Divergence Between Statistical Trends and Discussion Logic

Observation: The primary result is an HR of 1.42 (increased risk, though non-significant). Yet, the Discussion (Lines 221-237) extensively details the "tubal hypothesis" and "blood flow disruption," both of which support a protective effect (decreased risk).

Critique: There is a logical disconnect. The authors spend significant text explaining why hysterectomy should reduce risk, while their data suggests a trend towards increased risk.

Recommendation: The discussion needs to be balanced. The authors should address why their point estimate (1.42) differs from the protective effects (HR < 1.0) seen in older meta-analyses. Could this be due to Indication Bias? For example, women undergoing hysterectomy for endometriosis or adenomyosis might have an inherently higher baseline risk of ovarian cancer, which the Propensity Score Matching (PSM) might not have fully accounted for if pathology data was lacking.

**Response:** We appreciate Reviewer's insightful observation regarding the logical disconnect between our biological rationale (favoring protection) and point estimate (HR 1.42 suggesting increased risk). This tension is indeed central to interpreting our findings. To address this divergence, we have already added harmful mechanisms to both the Introduction and Discussion sections to achieve better balance. Your recommendation regarding indication bias was an excellent suggestion that we had not previously considered. We completely agree that women undergoing hysterectomy often have underlying conditions like endometriosis or adenomyosis, both established ovarian cancer risk factors that may not be fully balanced by claims-based propensity score matching (particularly without pathology data on disease severity).

The revised sentence now reads (lines 78-89): “Conversely, several biological mechanisms support the possibility of a null or even increased risk after simple hysterectomy. Simple hysterectomy leaves the fallopian tubes in situ, and accumulating pathologic and epidemiologic evidence indicates that many high-grade serous ovarian, fallopian tube, and primary peritoneal carcinomas arise from the distal fallopian tube epithelium rather than the ovarian surface itself[16, 17]. Therefore, unlike salpingectomy or salpingo-oophorectomy, simple hysterectomy does not remove the presumed primary site of origin for these tumors. In addition, hysterectomy-induced earlier menopause may lead to prolonged use of estrogen-only menopausal hormone therapy (MHT) in women without a uterus, and estrogen-only MHT has been associated with an increased risk of ovarian cancer in observational studies[18]. These competing mechanisms suggest that the net effect of hysterectomy on ovarian cancer risk is biologically uncertain and could plausibly range from risk reduction to no effect or even risk increase.” (lines 318-322): “Second, this discrepancy reflects indication bias: women requiring hysterectomy often have underlying conditions such as endometriosis or adenomyosis, both of which are recognized risk factors for ovarian cancer [34], but these factors may not be fully captured by claims-based propensity score matching.”

3. Statistical Power and Interpretation of "Inconclusive Findings"

Observation: The Abstract concludes findings are "inconclusive" due to wide Confidence Intervals (CI 0.79–2.56) and limited cases.

Critique: With 13,059 matched pairs, the study is relatively large, but the event rate is very low (incidence 18 vs 13 per 100,000). The extremely wide CI in the adnexal surgery subgroup (CI 0.251–8.977) renders that specific sub-analysis statistically useless.

Recommendation: Avoid over-interpreting the subgroup analyses. Acknowledging the "Type II Error" (false negative) probability is necessary. The authors should explicitly state in the Limitations that despite the large n, the study is underpowered for the specific outcome of ovarian cancer due to its rarity.

**Response:** We fully agree that our study is severely underpowered despite the large sample size, due to ovarian cancer's low incidence. In the revised Limitations section, we now explicitly state that the study was severely underpowered to detect anything but very large effect sizes, which fundamentally constrains the interpretability and conclusiveness of our findings. The revised sentence now reads (lines 347-349): “The most important limitation of this study is the limited number of ovarian cancer events, which resulted in wide confidence intervals and low statistical power.” (lines 352-355): “In particular, the study was severely underpowered to detect anything but a very large effect size, which fundamentally limits the conclusiveness of our findings and means that modest increases or decreases in risk cannot be ruled out.”

4. The "Temporal Shift" Argument

Observation: The Discussion (Lines 247-259) cites a meta-analysis suggesting a shift where post-2000 studies show a positive association (HR > 1).

Critique: This is the most interesting part of the literature review and aligns with the authors' own findings (HR 1.42).

Recommendation: Instead of focusing on the protective "tubal hypothesis," the authors should pivot the discussion to expand on this "temporal shift." Why are post-2000 studies showing increased risk? Is it better detection? Changes in surgical technique (e.g., sparing tubes in the past vs. opportunistic salpingectomy now)? Or is it that benign indications for hysterectomy (like endometriosis) are now better understood as risk factors for ovarian cancer?

**Response:** We especially thank Reviewer for emphasizing the temporal shift observation, which indeed aligns closely with our findings. In the expanded Discussion, we now hypothesize several explanations:

The revised sentence now reads (lines 315-322): “Several plausible explanations may account for this finding. First, our findings align with the 'temporal shift' observed in a 2013 meta-analysis , which reported protective effects (pooled RR 0.70) in pre-2000 studies but increased risk (RR 1.18) in post-2000 studies. This pattern may reflect improved diagnostic specificity in distinguishing ovarian cancer. Second, this discrepancy reflects indication bias: women requiring hysterectomy often have underlying conditions such as endometriosis or adenomyosis, both of which are recognized risk factors for ovarian cancer[34], but these factors may not be fully captured by claims-based propensity score matching.”

Minor Comments
ICD-10 Code Specificity: The abstract mentions "C56.xx" (Line 35). Does this exclude Borderline Ovarian Tumors (BOT)? Inclusion of BOTs can inflate survival and incidence rates. Please clarify if the study is strictly invasive epithelial ovarian cancer.

**Response:** Thank you for highlighting the issue of ICD-10 specificity.

In this study, incident ovarian cancer was defined as ≥3 medical visits with an ICD-10 diagnosis code C56.xx (primary or secondary diagnosis), which corresponds to malignant neoplasm of the ovary. Borderline ovarian tumors are typically coded under ICD-10 “neoplasm of uncertain behavior of ovary” (e.g., D39.1 and its subcodes) rather than C56, and therefore BOT would not be counted as outcomes under our C56.xx-based definition. Because our outcome definition relies on C56.xx claims codes (and not pathology/histology), we cannot further distinguish invasive epithelial ovarian cancer from other malignant ovarian tumor histologies within C56.xx in this dataset.

■Materials and Methods (Outcomes)

(Section 2.3 Outcomes; lines 144) Added one clarifying sentence.

Added text (lines 144-146): “Because the endpoint was defined using ICD-10 C56.xx claims codes, it captures malignant ovarian neoplasms coded as C56 and does not include borderline ovarian tumors typically coded as D39.1.”

Follow-up Duration: The median follow-up is 11.5 years. The discussion (Lines 283-285) mentions that protective effects might be seen after 20 years. The authors should explicitly recommend a longer follow-up study as a future direction.

**Response:** We thank Reviewer #3 for this important observation regarding our follow-up duration. We agree that the median follow-up of 11.5 years [10-13.4] may be insufficient to capture potential late-emerging protective effects of hysterectomy on ovarian cancer risk.

In response, we have added an explicit recommendation for future studies with longer follow-up in the Discussion section (lines 374-377): “Future prospective studies with ≥20-year follow-up are needed to assess whether protective effects of hysterectomy on ovarian cancer risk emerge only after longer latency periods, as suggested by prior analyses showing risk reduction primarily among women ≥20 years post-hysterectomy”

Terminology: In Line 41, the term "adnexal surgery" is vague. Use precise terms like "Unilateral Salpingo-Oophorectomy" or "Bilateral Salpingectomy" throughout the manuscript.

**Response:** Thank you for noting that “adnexal surgery” is vague and recommending precise surgical terms. We agree in principle. However, in the Korean NHIS claims/procedure coding used in this study, laterality (unilateral vs bilateral) is not consistently identifiable, and therefore we cannot accurately label procedures as unilateral vs bilateral salpingo-oophorectomy/salpingectomy. To avoid incorrect terminology, we standardized the wording to “concomitant adnexal surgery (salpingectomy and/or oophorectomy identified by procedure codes)” and added an explicit statement explaining that laterality cannot be determined from the NHIS procedure codes used in this analysis.

■Methods (define term)

(Line 154) The manuscript currently states that “hysterectomy or adnexal surgery (recorded using surgery codes)” was included as a variable.

Revised text (lines 159-160): “Concomitant adnexal surgery was defined using NHIS procedure codes indicating salpingectomy and/or oophorectomy performed at the time of hysterectomy.”

■Methods (laterality limitation)

Added text (lines 161-162): “Because laterality is not reliably captured in the NHIS procedure codes available for this study, we could not distinguish unilateral from bilateral salpingectomy/oophorectomy.”

■Global terminology change

(Throughout manuscript where applicable, including Abstract/Results/Discussion) Replaced the vague phrase “adnexal surgery” with: “concomitant adnexal surgery (salpingectomy and/or oophorectomy, as identified by procedure codes).”

Typo/Formatting: Line 215 states "HR 1.421" and Line 41 says "1.42". Please ensure consistent decimal places (2 decimal places is standard for HR reporting in abstracts).

**Response:** We thank Reviewer for noting the inconsistency in hazard ratio decimal places. We agree that two decimal places is the standard for HR reporting in abstracts and main text. We have standardized all HR values to two decimal places throughout the manuscript.

1.	Yang, M.S., et al., *Validation of Cancer Diagnosis Based on the National Health Insurance Service Database versus the National Cancer Registry Database in Korea.* Cancer Res Treat, 2022. **54**(2): p. 352-361.
